# Expression of concern: Microgel-stabilised non-aqueous emulsions

**DOI:** 10.1039/d5ra90043c

**Published:** 2025-04-14

**Authors:** Amro K. F. Dyab, Ayman M. Atta

**Affiliations:** a Chemistry Department, Faculty of Science, Minia University Minia Egypt amr.diab@mu.edu.eg; b Department of Chemistry, College of Science, King Saud University P. O. Box 2455 Riyadh 11451 Saudi Arabia

## Abstract

Expression of concern for ‘Microgel-stabilised non-aqueous emulsions’ by Amro K. F. Dyab *et al.*, *RSC Adv.*, 2013, **3**, 25662–25665, https://doi.org/10.1039/C3RA45263H.

*RSC Advances* is publishing this expression of concern in order to alert readers that concerns have been raised regarding the integrity of the TEM data in Fig. 1.

An expression of concern will continue to be associated with the article until a conclusive outcome is reached.

Laura Fisher

4th April

Executive Editor, *RSC Advances*

